# CSF-1-induced DC-SIGN^+^ macrophages are present in the ovarian endometriosis

**DOI:** 10.1186/s12958-022-00901-w

**Published:** 2022-03-08

**Authors:** Li Xiaocui, Hong Wei, Cai Yunlang, Zheng Zhenzhen, An Min

**Affiliations:** 1 Department of Obstetrics and Gynecology, Shanghai First Maternity and Infant Hospital, Tongji University School of Medicine, Shanghai, 201204 P.R. China; 2 Department of Obstetrics and Gynecology, Medical School, Zhongda Hospital, Southeast University, Nanjing, 210009 China

**Keywords:** Endometriosis, DC-SIGN^+^ macrophages, CSF-1, Treg cells

## Abstract

**Background:**

Researchers have found that macrophages are the predominant cells in the peritoneal fluid (PF) of endometriosis patients. CSF-1 has been found to accumulate in the lesions and PF of endometriosis patients, and CSF-1 induces THP-1-derived macrophages to polarize toward a CD169^+^ DC-SIGN^+^ phenotype. Does the cytokine CSF-1 induce monocytes to differentiate into macrophages with a DC-SIGN^+^ phenotype in endometriosis?

**Methods:**

The level of CSF-1 in the endometrium of control subjects, and the eutopic, and ectopic endometrium of endometriosis patients was evaluated by real-time polymerase chain reaction (qRT–PCR) and was determined by enzyme-linked immunosorbent assay (ELISA) in the PF of control and endometriosis patients. CSF-1 expression was examined with a MILLIPLEX MAP Mouse Cytokine/Chemokine Magnetic Bead Panel. DC-SIGN^+^ macrophages were detected by immunohistochemical staining of tissues and flow cytometric analysis of the PF of control subjects (*N* = 25) and endometriosis (*N* = 35) patients. The phenotypes and biological activities of CSF-1 -induced macrophages were compared in an in vitro coculture system with peripheral blood lymphocytes from control subjects.

**Results:**

In this study, we found that the proportion of DC-SIGN^+^ CD169^+^ macrophages was higher in the abdominal immune microenvironment of endometriosis patients. CSF-1 was primarily secreted from ectopic lesions and peritoneum in mice with endometriosis. In addition, CSF-1 induced the polarization of macrophages toward a DC-SIGN^+^ CD169^+^ phenotype; this effect was abolished by the addition of an anti-CSF-1R antibody. CSF-1 induced the generation of DC-SIGN^+^ macrophages, leading to a depressed status of peripheral blood lymphocytes, including a high percentage of Treg cells and a low percentage of CD8^+^ T cells. Similarly, blockade with the anti-CSF-1R antibody abrogated this biological effect.

**Conclusions:**

This is the first study on the role of DC-SIGN^+^ macrophages in the immune microenvironment of endometriosis. Further study of the mechanism and biological activities of CSF-1-induced DC-SIGN^+^ macrophages will enhance our understanding of the physiology of endometriosis.

**Graphical Abstract:**

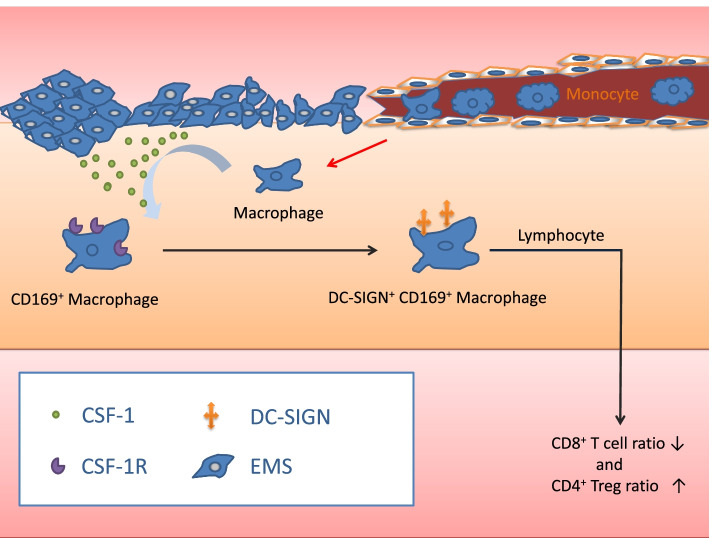

## Background

Endometriosis (EMS) is a common but complicated disease that causes chronic pelvic pain, dyspareunia and infertility in 6–10% of women of reproductive age [1]. Although the retrograde menstruation hypothesis has been widely accepted [2], in recent years, some studies have suggested that retrograde menstruation is a precondition of EMS development and that the abnormal metabolism of hormones is a secondary condition; however, immune dysfunction is a cause of EMS [3].

A number of studies have been conducted on peritoneal immunity in EMS, but many have been limited to a description of immune cell dysfunction [4–6]. Some experiments suggested the depressed state of peritoneal immunity in EMS patients [7–9]. With the widespread study of the immune system in the abdominal microenvironment, the profound role of macrophages in the pathological physiology of EMS is becoming clear [10]. Crucial functions of macrophages, such as stabilizing tissue structure, driving injury, and regulating the immune microenvironment, have been observed [11]. Our previous studies suggested that macrophages are involved in the progression of EMS [12]. In addition, along with the high number of macrophages, the proportions of Treg and Th1 cells in the peritoneal fluid of mice with EMS were found to be increased in a previous in vivo study [13].

Peritoneal immunity is complex and powerful, and it is surprising that the ectopic endometrium survives during the menstrual cycle. Colony stimulating factor-1 (CSF-1), also called macrophage colony-stimulating factor (M-CSF), is a key cytokine that induces monocytes to differentiate into CD169^+^ macrophages [14]. CSF-1 and its receptor CSF-1R (M-CSFR or CD115) are diffusely expressed and have frequently been reported in the context of EMS [15, 16]. A study showed that the expression of CSF-1 was increased in the PF of EMS patients [17]. According to the literature, CD169^+^ macrophages are found in various diseases and indirectly affect Treg cells and CD8^+^ T cells [18, 19]. In a study of transplantation tolerance, CD169^+^ DC-SIGN^+^ macrophages were found to interact with lymphocytes to maintain transplant survival [20]. Many studies have proven that FOXP3^+^ Treg cells play an important role in immunoregulation and immunosuppression [21, 22]. FOXP3^+^ Treg cells secrete cytokines, such as CCL22, CCL17, transforming growth factor-1 (TGF-1), interleukin (IL)-1, tumor necrosis factor (TNF), IL-8 and vascular endothelial growth factor (VEGF), while cooperating with other immune cells to promote the progression of EMS [23].

Szyllo K, et al. determined that a small percentage of CD8^+^ T cells are related to EMS [24]. A study showed that CD169^+^ macrophages had a profound effect on the cellular immunologic response [25]. Moreover, CD169 + macrophages were found to induce the polarization of Treg cells and regulate the immune response [26]. Therefore, the critical aim was to identify the common targets through which macrophages communicate with Treg cells and CD8^+^ T cells.

DC-specific ICAM-3-grabbing nonintegrin (DC-SIGN or CD209) on CD169^+^ macrophages is a key target in the regulation of depressed immunity. This molecule was first described by Geijtenbeek TBH, et al., in a study about a dendritic cell-specific HIV-1-binding protein that enhances trans-infection of T cells [27]. Mechanistically, these authors explained that DC-SIGN is an important molecule that participates in the process by which DCs interact with CD4^+^ T cells [28]. Soilleux E. J. et al. proved that some specific macrophages, such as those in the placenta and decidua, expressed DC-SIGN [29]. Marie Larsson et al. observed that DC-SIGN^+^ DCs regulated CD8^+^ T cells [30]. Recently, some studies have suggested that DC-SIGN is a type of regulatory receptor on DCs and macrophages that alters the function of macrophages [31]. Overall, the aim of our study was to prove that CSF-1 induces monocytes to differentiate into macrophages with a CD169^+^ DC-SIGN^+^ phenotype in endometriosis and that this macrophage phenotype can influence the differentiation of T cells.

## Methods

### Experimental design

CSF-1 was evaluated by qRT–PCR in the tissues of control subjects and endometriosis patients, and was determined by ELISA in the PF of control and endometriosis patients. Then, CSF-1 expression was examined with a MILLIPLEX MAP Mouse Cytokine/Chemokine Magnetic Bead Panel in the EMS model. DC-SIGN^+^ macrophages were detected by immunohistochemical staining of tissues and flow cytometric analysis of the PF of control subjects and endometriosis patients. The phenotypes and biological activities of CSF-1 -induced DC-SIGN^+^ macrophages were compared using flow cytometric analysis in an in vitro coculture system with peripheral blood lymphocytes.

### Tissue and sample collection

For immunohistochemical staining, 39 women with EMS (American Fertility Society classification as endometriosis Stage III ( *n* = 30) or Stage IV (*n* = 9)) who were diagnosed by clinical pathologists at Shanghai First Maternity and Infant Hospital Affiliated with Tongji University School of Medicine, between June 2019 and May 2020 were recruited. Eutopic endometrial samples were obtained from women with EMS (*n* = 17). For the control samples, endometrial tissue samples were collected by curettage from 11 women who exhibited fallopian tube blockage and previously needed contraception by fallopian tube ligation but now needed tubal reversal, and did not have any clinical indication or history of adenomyosis or endometriosis. PF was collected, avoiding contamination with blood, from women with advanced-stage EMS (*n* = 35) and from control subjects (*n* = 25) during laparoscopic surgery. All the enrolled patients in the control group were confirmed by laparoscopy to have no endometriotic lesions, and all had a histological diagnosis of mature ovarian teratoma composed of epidermis only (*n* = 20) or benign cyst of the mesosalpinx (*n* = 5). The clinical characteristics of the patients and controls are summarized in Table 1.

**Table 1:**
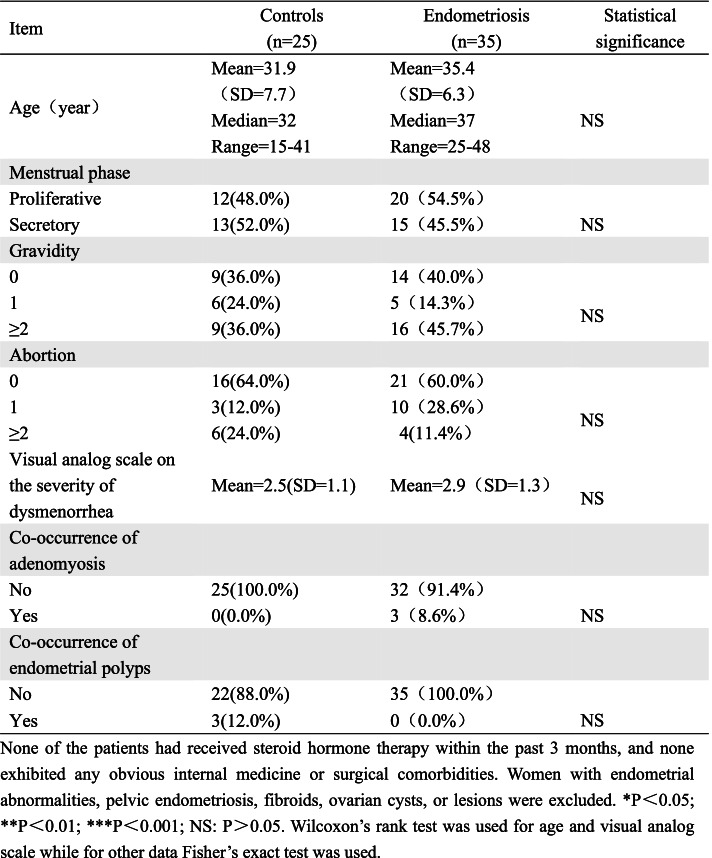
Demographic characteristics of patients with endometriosis and controls

None of the patients had received steroid hormone therapy within the past 3 months, and none exhibited any obvious internal medicine or surgical comorbidities. Women with endometrial abnormalities, pelvic endometriosis, fibroids, ovarian cysts, or lesions were excluded. *P<0.05; **P<0.01; ***P<0.001; NS: *P*>0.05. Wilcoxon’s rank test was used for age and visual analog scale while for other data Fisher’s exact test was used

### Cell lines, cytokines and animals

The acute monocytic leukaemia (THP-1) cell line was a gift from the Cryomedicine Lab at Qilu Hospital of Shandong University. Recombinant human M-CSF was purchased from R&D Systems (Minnesota, USA). C57BL/6 mice were purchased from Shanghai SLAC Laboratory Animal Co., Ltd., at 4 weeks of age.

### Real-time polymerase chain reaction

To test the gene expression level of CSF-1 in the endometrium of control subjects (*N* = 11) and the eutopic endometrium (*N* = 17) and ectopic (*N* = 39) endometrium of endometriosis patients, we conducted PCR. Total RNA was extracted from 0.2 × 0.2 × 0.1 cm tissue fragments using TRIzol Reagent (10,296,010, Invitrogen, USA) according to the product manual. RNA (0.5 μg) was reverse transcribed into cDNA with ReverTra Ace Quantitative PCR (qPCR) RT Master Mix with gDNA Remover (Code No. FSQ-301, TOYOBO, Osaka Japan). Each 20 μl PCR mixture contained 1 × SYBR Green PCR Master Mix (TOYOBO, Osaka Japan), 30 ng of cDNA and 300 nM each specific primer from Sangon Biotech (Shanghai) Co., Ltd. Quantitatie PCR was performed on an Applied Biosystems StepOnePlus RT–PCR System (Applied Biosystems, USA). Three separate experiments were performed on different cultures, and each sample was assayed in triplicate. The mean was calculated to determine the mRNA levels by quantitative real-time polymerase chain reaction (RT–PCR) analysis using the ABI StepOnePlus RT–PCR System (Applied Biosystems). The primers used to amplify CSF-1 were 5'-GCTGCTTCACCAAGGATTATG-3' (sense) and 5'-GGGTCACTGCTAGGGATG-3' (antisense), and the gene expression levels in each group was normalized to that of glyceraldehyde 3-phosphate dehydrogenase (GAPDH), which was assessed with the following primers: 5'-AGCCACATCGCTCAGACA-3' (sense) and 5'-GCCCAATACGACCAAATCC-3' (antisense). The mean relative gene expression levels were determined, and differences in expression were calculated using the 2^−△△Ct^ method.

### Establishment of the EMS model

All mice were maintained under specific pathogen-free (SPF) conditions. All procedures were approved by the Animal Care and Use Committee of Tongji University (Shanghai, China). The estrous stage was monitored daily by obtaining a vaginal smear every morning for 2 weeks, and mice with a normal estrous cycle were used in the following experiments. Endometriosis was established by intraperitoneal injection of endometrial segments. Briefly, donor mice were sacrificed, and the uteri were isolated and collected in a Petri dish containing phosphate-buffered saline (PBS). The isolated uterine horns were treated identically, including longitudinal splitting with a pair of scissors, isolation of endometrial tissue and gentle disruption to generate small, uniform fragments of approximately 1 mm. Then, these fragments were intraperitoneally injected into recipient mice through a 1 ml syringe with a 25 G needle. Equal amounts of endometrial fragments from one donor mouse were injected into two recipient mice. Specifically, 15 endometrial segments in 200 μl of sterile PBS were injected into each recipient mouse. However, mice in the control groups only received injections of 200 µL of sterile PBS. Finally, ten mice from the endometriosis and control groups were sacrificed 42 days after the model was established.

### Analysis of CSF-1 with a MILLIPLEX MAP Mouse Cytokine/Chemokine Magnetic Bead Panel

A MILLIPLEX® MAP kit for analysis was used to evaluate tissue (uterus, peritoneum, and ectopic lesions) cytokine production (CSF-1) and release in the PF of EMS mice, in accordance with the manufacturer’s instructions. In addition, the plate was analyzed on a Luminex 200 system. The sample data were analyzed using xPONENTLX200 software (Madison, WI, USA).

### Immunohistochemical staining

To detect DC-SIGN^+^, CD169^+^, and CSF-1R^+^ macrophages and FOXP3^+^ T cells in the tissues of control subjects (*N* = 25) and endometriosis patients (*N* = 35), we conducted immunohistochemical staining. For each sample, 4 μm thick sections were dewaxed and rehydrated in ethanol and water. Antigen retrieval was performed in citrate buffer (pH 6.0, 15 min), and endogenous peroxidase activity was quenched by incubation in 3% hydrogen peroxide. The tissue sections were incubated overnight at 4 °C with rabbit primary antibodies against human CD169 (ab183356, Abcam, Cambridge, UK; 1:100), CSF-1R (ab183316, Abcam, Cambridge, UK; 1:100), and DC-SIGN (ab5715, Abcam, Cambridge, UK; 1:50) and mouse monoclonal antibodies against human CD68 (ab955, Abcam, Cambridge, UK; 1:200) and FOXP3 (ab20034, Abcam, Cambridge, UK; 1:50). A secondary antibody kit (CWBIO, Beijing, China) was utilized to label the primary antibodies. The sections were counterstained with hematoxylin, dehydrated in ethanol and xylene, and mounted in PermountTM mounting medium. Images were acquired using a microscope (BX53, Olympus, Tokyo, Japan), which was fitted with a digital camera (cellSens Standard, Olympus). A series of five images was randomly selected from six sections per tissue sample. Positive cells in images acquired at 400 × magnification were counted manually by two independent investigators blinded to the treatment groups. Cells were counted in at least five different areas in ectopic lesions, and data were plotted as the percentage of positive cells relative to the total number of cells, as previously described [32]. The percentage of positive cells was calculated by counting positive cells among a total of 200 cells in three separate fields.

### Macrophage induction and culture

THP-1 cells were induced to differentiate into macrophages in accordance with a previously reported method [33]. Cells cultured in 24-well plates were stimulated by adding 100 ng/ml phorbol 12-myristate 13-acetate (PMA; Sigma, USA) for 48 h and were than washed three times with PBS to eliminate the effect of PMA.

### Cellular Immunofluorescence Analysis

To detect the DC-SIGN^+^ and CD169^+^ macrophages derived from THP-1 cells induced by CSF-1 and blocked with CSF-1R, we conducted a cellular immunofluorescence analysis. We treated the differentiated macrophages with CSF-1 (50 ng/ml) and cultured them for 48 h. For confirmation, we cultured the differentiated macrophages with RPMI 1640 medium (Gibco, China) containing anti-CSF-1R (Abcam, UK, 1:20) for 2 h and then added CSF-1 (R&D Systems, USA, 50 ng/ml) to the medium for 48 h. Cellular immunofluorescence staining was performed as previously described [34]. The target macrophages were washed twice with PBS, fixed with 4% paraformaldehyde (pH 7.0) for 30 min, permeabilized with 0.1% Triton X-100 (Sigma) for 10 min, blocked with 10% normal goat serum for 1 h, and incubated overnight with rabbit anti-human CD169 (ab183356, Abcam, UK; 1:100) or anti-DC-SIGN (ab5715, Abcam, UK; 1:50) antibodies and a mouse monoclonal antibody against human CD68 (ab955, Abcam, UK; 1:200) at 4 °C. The cells were then washed with PBS three times and incubated at room temperature with a DyLight 488-conjugated donkey anti-rabbit secondary antibody (1:400; Abcam, UK) or a DyLight 594-conjugated donkey anti-goat secondary antibody (1:400; Abcam, UK) for 1 h. After the plates were washed, cell nuclei were counterstained with fluorescent mounting medium with 4’,6-diamidino-2-phenylindole (Abcam, UK).

### Coculture of peripheral blood mononuclear cells (PBMCs) with THP-1-derived macrophages

To check the influence of DC-SIGN^+^ CD169^+^ macrophages derived from THP-1 cells on T cells, we cocultured PBMCs with THP-1-derived macrophages. PBMCs were prepared as previously described [35]. Volunteers were recruited from among healthy women without dysmenorrhea (*n* = 7) and with a regular menstrual cycle. PBMCs were isolated from 15 ml of venous blood using Ficoll-Cardiografin (Tian Jin Hao Yang Biological Manufacture, China). After centrifugation, PBMCs were collected from the interphase layer and washed four times with PBS. For coculture, PBMCs were suspended in RPMI 1640 medium supplemented with FBS at a concentration of 1 × 10^6^ cells/ml. The coculture system simulated the lesion site in the human body. Transwell membranes (Corning, USA) were used to allow the exchange of macrophage secretions with PBMCs. First, Transwell membranes were inserted into a 24-well plate, and the upper chamber was preseeded with macrophages derived from THP-1 cells. In addition, 100,000 PBMCs were counted and seeded on the membrane. Second, two groups of macrophages derived from THP-1 cells were treated with a rabbit anti-human DC-SIGN (Abcam, UK; 1:50) or CSF-1R (Abcam, UK; 1:20) antibody and with CSF-1 (50 ng/ml) for 48 h. Third, we cocultured the differentiated macrophages with PBMCs, as shown in (Fig. 5A). Then, the Transwell plates were placed in a 5% CO_2_ incubator at 37 °C for 2 days. After incubation, the cocultured PBMCs were collected.

### Flow cytometry

The remaining peritoneal cells collected from the controls and the patients were divided into two equal parts for analysis by flow cytometry (FCM). To determine the percentages of the DC-SIGN^+^ CD169^+^ macrophage subset, cells in the PF at a density of 0.5 × 10^5^ cells/tube were used and stained in duplicate in the dark at 4 °C for 30 min with the following antibodies: APC-anti-human CD209 (clone: 9E9A8, Biolegend, USA), FITC-anti-human CD45 (clone: 2D1, Biolegend, USA), PerCP-Cy5.5-anti-human CSF-1R (clone: 9-4D2-1E4, Biolegend, USA) and PE-anti-human CD169 (clone: 7–239, Biolegend, USA). Fluorophore-conjugated and isotype-matched antibodies served as the negative controls. The proportions of Treg cells and CD8^+^ T cells in the PBMCs collected from the coculture system were determined using FCM. PBMCs at a density of 0.5 × 10^5^ cells/tube were fixed, permeabilized, and stained in duplicate with the following antibodies: APC-anti-human FoxP3 (clone: 3G3, Assay Genie, Ireland), FITC-anti-human CD45 (clone: 2D1, Biolegend, USA), PerCP-Cy5.5-anti-human CD8 (clone: SK1, Biolegend, USA), and PE-anti-human CD4 (clone: SK3, Biolegend, USA). The proportions of distinct subpopulations were eventually characterized on a BD FACSCalibur (BD, USA). The resulting data were examined using FlowJo software. A representative graph from three or more repeated experiments is shown. Positive gates were set separately for each sample type and label and were set to include less than 1% of the acquired events in the unstained sample.

### Enzyme-linked immunosorbent assay (ELISA)

First, an aliquot of each preparation was kept for protein estimation using a Bradford protein concentration assay kit (P0006C) from Beyotime. The CSF-1 levels in the PF of EMS patients and controls were quantified using each ELISA kit (R&D Systems), according to the manufacturer’s instructions. The absorbance of the streptavidin–horseradish peroxidase colorimetric reaction product was measured with a microplate reader capable of measuring the absorbance at 450 nm, with the correction wavelength set at 540 nm or 570 nm. The optical density values were compared with those of serial dilutions of recombinant human CSF-1 as a standard. The relevant concentration was calculated, and standard curves were generated.

### Statistical analysis

All statistical analyses were completed using Prism software, version 5 (GraphPad, San Diego, CA, USA), and the measurement data are presented as the mean ± SD values. Statistical significance was assessed using Student’s t test for univariate analyses of two sets of data by one-way or two-way analysis of variance (ANOVA) when multiple comparisons were necessary. *P* < 0.05 was considered to indicate significance.

## Results

### The proportion of DC-SIGN^+^ macrophages was higher in endometriosis patients

**Fig. 1:**
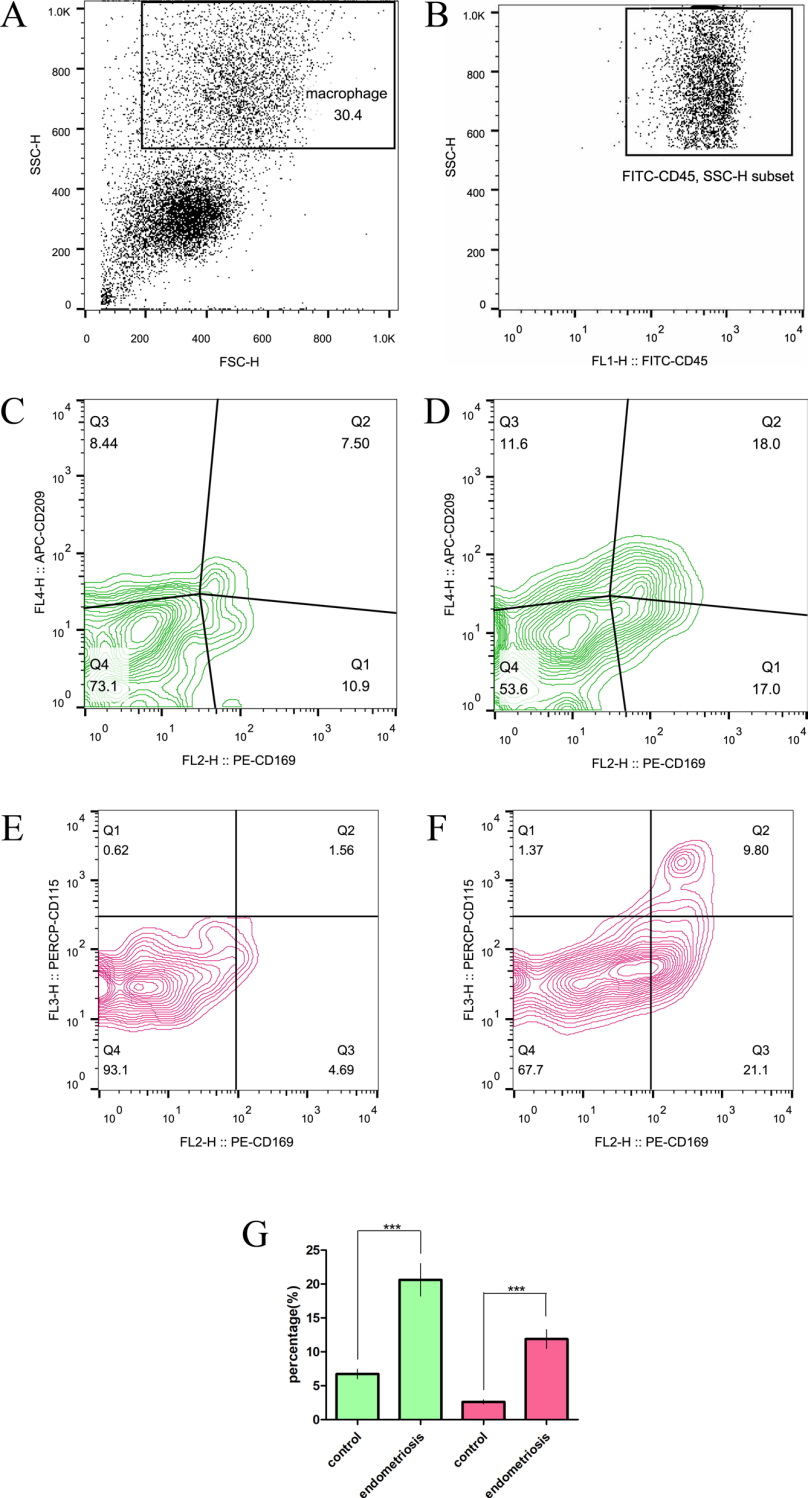
There were more CD169^+^ CD115^+^ macrophages and CD169^+^ CD209^+^ macrophages in the PF of endometriosis patients than in that of control subjects. **A** Morphological cytograms showing the FSC (forward scatter) and SSC (side scatter) parameters of peritoneal cells in PF. **B** The monocyte/macrophage population gated on CD45 and SSC. **C** Gating of CD169^+^ CD209^+^ macrophages in the PF of controls. **D** Gating CD169^+^ CD209^+^ of macrophages in the PF of the endometriosis patients. **E** Gating of CD169^+^ CD115^+^ macrophages in the PF of controls. **F** Gating of CD169^+^ CD115^+^ macrophages in the PF of endometriosis patients. **G** Differences between groups were analyzed by Student’s t test (controls, *n* = 25; endometriosis patients, *n* = 35). The values are expressed as the means ± SDs. ***P ≤ 0.001

We collected peritoneal cells from the PF of patients for flow cytometry and performed a series of analyses (Fig. 1). Macrophages express higher levels of the CD45 antigen, which is exclusively expressed on all nucleated cells of the hematopoietic system, and the different proportions of the monocyte/macrophage populations in the peritoneal cavity can be further confirmed in morphological cytograms showing the FSC (forward scatter) and SSC (side scatter) parameters obtained by flow cytometry [36] (Fig. 1A-B). We analyzed the coexpression of DC-SIGN and CD169 by macrophages (Fig. 1C-D) and the coexpression of CSF-1R and CD169 by macrophages (Fig. 1E-F). From the data analysis, we found that in the PF of endometriosis patients, the coexpression of DC-SIGN and CD169 at macrophages was greater than that in the control group, and the coexpression of CSF-1R and CD169 by macrophages was greater than that in the control group (Fig. 1G).

**Fig. 2:**
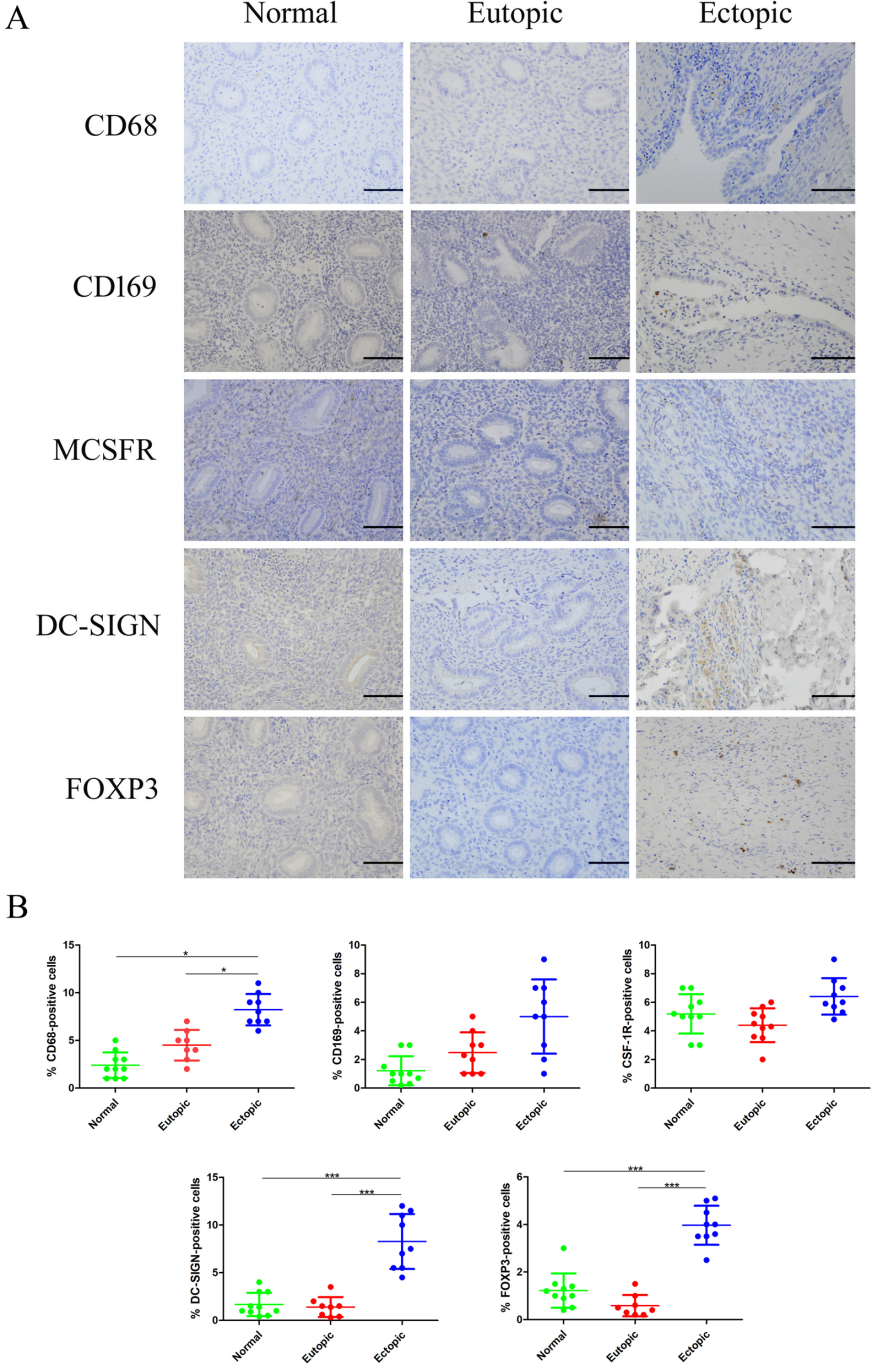
Macrophages and Treg cells in the ectopic and eutopic endometrium of endometriosis patients and the normal endometrium. **A** The expression of CD68, CD169, CSF-1R, and DC-SIGN in macrophages, and the expression of FOXP3 in Treg cells in all tissues of the normal endometrium, eutopic endometrium of endometriosis patients, and ectopic endometrium of endometriosis patients. Representative images are shown at 400 × magnification. **B** The comparison between the proportion of macrophages and Treg cells in tissues of the normal endometrium, eutopic endometrium of endometriosis patients, and ectopic endometrium of endometriosis patients. The data are presented as the means ± SDs. Statistical analyses were performed by one-way analysis of variance (eutopic = 17; ectopic = 17; normal = 11), and significant differences are indicated as *P ≤ 0.05 and ***P ≤ 0.001

We also conducted immunohistochemical staining and performed a series of analyses of the immune cells detected in all the tissues (Fig. 2A). In ovarian endometriotic lesions, we observed significantly more CD68^+^ macrophages and DC-SIGN^+^ macrophages than in the normal endometrium and in the eutopic endometrium of endometriosis patients. Additionally, in ovarian endometriotic lesions, we observed significantly higher numbers of FOXP3^+^ T cells than in the normal endometrium or in the eutopic endometrium of the endometriosis patients. In contrast, concerning CD169^+^ macrophages and CSF-1R^+^ macrophages, there were no significant differences between the ovarian endometriotic lesions, the normal endometrium and the eutopic endometrium of the endometriosis patients (Fig. 2B).

### CSF-1 levels are elevated in peritoneal fluid and ectopic endometrium from women with endometriosis

**Fig. 3:**
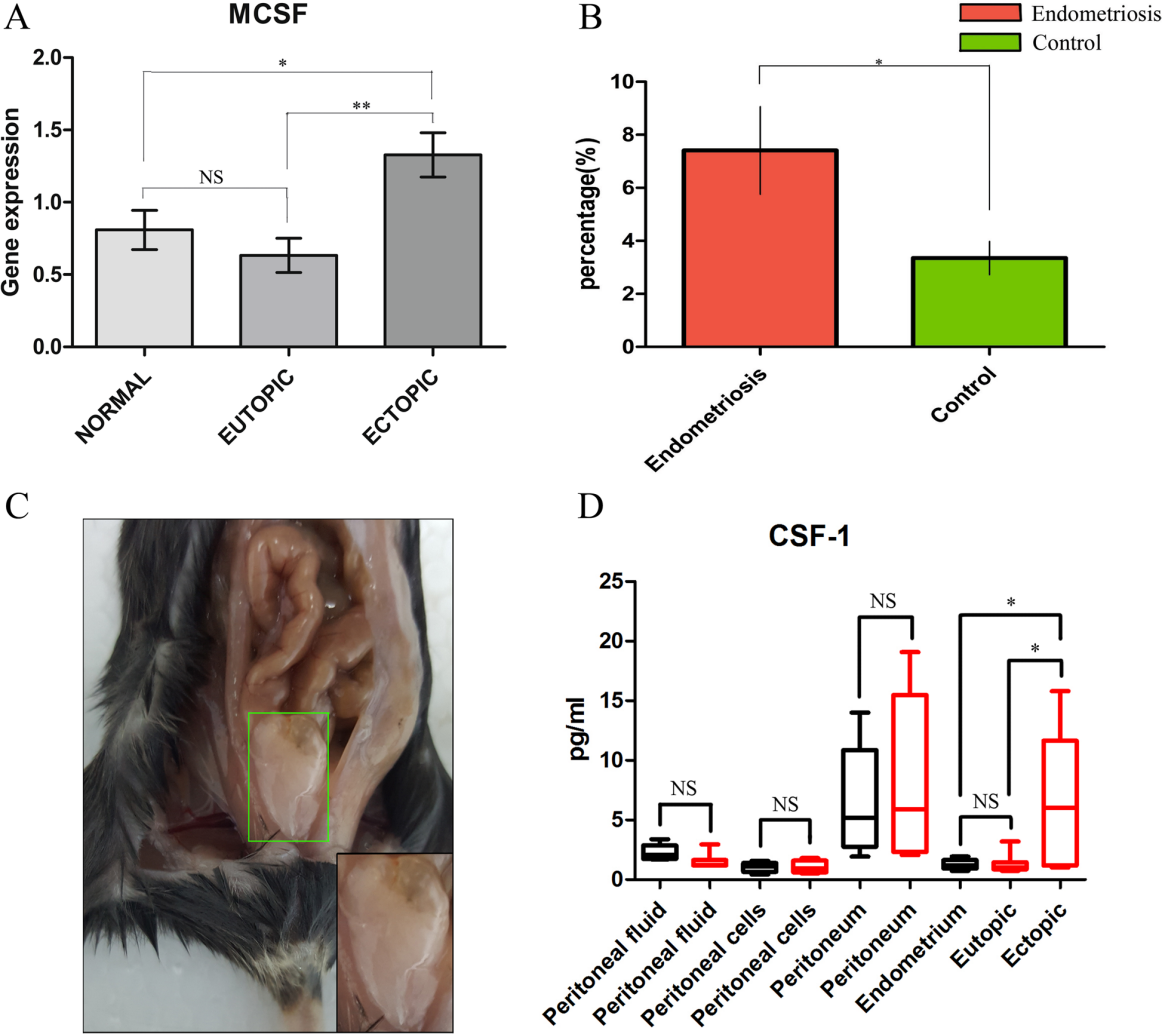
CSF-1 levels are elevated in peritoneal fluid and ectopic endometrium from women with endometriosis and in ectopic lesions and the peritoneum of mice with endometriosis. **A** The gene expression of CSF-1 in the normal endometrium, eutopic endometrium of endometriosis patients and ectopic endometrium of endometriosis patients, as detected by RT–PCR. **B** The percentage of the cytokine CSF-1 amoung total protein, as detected by ELISA, in the PF of the control and endometriosis groups. **C** The EMS model and ectopic lesions in the EMS model. **D** The level of the cytokine CSF-1 in the PF, peritoneal cells of the PF, peritoneum, normal endometrium, eutopic endometrium, and ectopic endometrium in the control and EMS model was analyzed by the MILLIPLEX MAP Mouse Cytokine/Chemokine Magnetic Bead Panel. The data are presented as the means ± SDs. Statistical analyses were performed by Student’s t test (controls, *n* = 25; endometriosis patients, *n* = 35; normal mice, *n* = 9; EMS mice, *n* = 9) and one-way analysis of variance (eutopic = 17; ectopic = 17; normal = 11). Significant differences are indicated as *P ≤ 0.05 and **P ≤ 0.01

We observed a relative increase in CSF-1 gene expression in ectopic endometrial tissue compared with the paired eutopic endometrial tissue and normal endometrial tissue (Fig. 3A). Our data demonstrated that peritoneal fluid from women with endometriosis contained a significantly higher percentage of CSF-1 than that from control subjects (Fig. 3B). In the EMS model, we found that cytokine production and CSF-1 levels were elevated in the ectopic lesions and peritoneum of mice with endometriosis, although there was no difference between the peritoneal fluid, peritoneal cells, and endometrium of normal mice and mice with endometriosis (Fig. 3C-D). Although there were high levels of CSF-1 in the peritoneum of normal mice and mice with endometriosis, the level of CSF-1 in the peritoneum of mice with endometriosis was higher than that in normal mice; however, there was no statistically significant difference (Fig. 3D).

### CSF-1 induced macrophages to acquire a DC-SIGN^+^ phenotype, which could be abolished by the addition of anti-CSF-1R

**Fig. 4:**
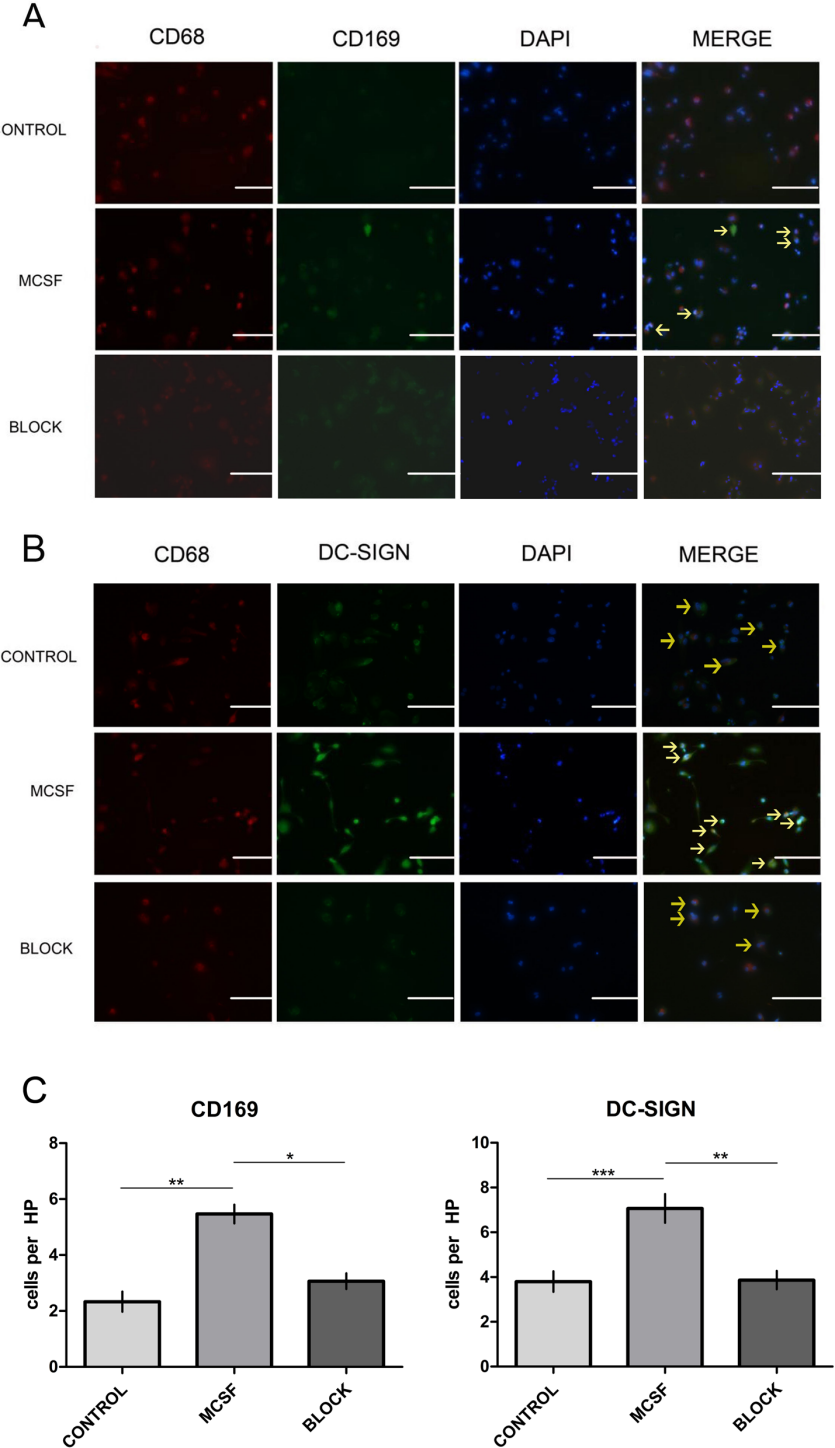
CSF-1 induces macrophages to differentiate into a DC-SIGN^+^ phenotype, and this effect can be abolished by the addition of an anti-CSF-1R antibody. **A** CD169 expression in uninduced macrophages, macrophages induced by CSF-1 (MCSF) and macrophages induced by CSF-1 (MCSF) after blockade with the anti-CSF-1R antibody. **B** DC-SIGN expression in uninduced macrophages, macrophages induced by CSF-1 (MCSF) and macrophages induced by CSF-1 (MCSF) after blockade with the anti-CSF-1R antibody. Representative images are shown at 200 × magnification (*n* = 5). **C** The comparison between the numbers of CD169^+^ macrophages and DC-SIGN^+^ macrophages per high -power field (per HP) in the control groups, MCSF groups, and blockade groups in the in vitro experiment. The data are presented as the means ± SDs. Statistical analyses were performed by one-way analysis of variance and significant differences are indicated as *P ≤ 0.05, **P ≤ 0.01, and ***P ≤ 0.001

The successful induction of macrophages from THP-1 cells was monitored by evaluating the characteristic CD68-positive cells (Fig. 4). A significant increase in CD169^+^ macrophages was observed in the CSF-1-treated group, while in this model, immunofluorescence staining showed that, after the CSF-1 antagonist anti-CSF-1R was added, the expression of CD169 was low (Fig. 4A). Similarly, CSF-1-treated macrophages demonstrated significant upregulation of DC-SIGN, but after the anti-CSF-1R antibody was added, the expression of DC-SIGN was decreased (Fig. 4B).

### CSF-1 induced the generation of DC-SIGN^+^ macrophages, leading to a suppressive phenotype of peripheral blood lymphocytes, while blockade with anti-CSF-1R abrogated this biological effect

**Fig. 5:**
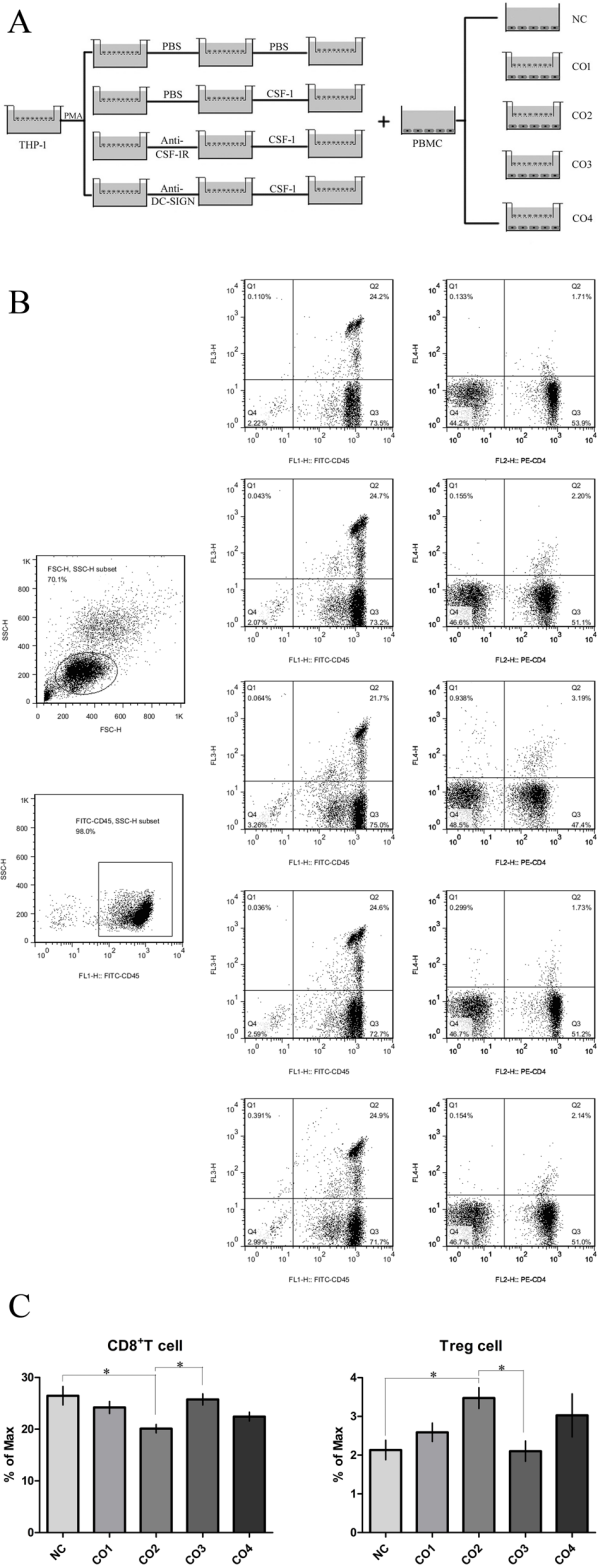
CSF-1- induced the differentiation of DC-SIGN^+^ macrophages, leading to a suppressive phenotype of peripheral blood lymphocytes, while anti-CSF-1R abrogated this effect. **A** The coculture system of treated macrophages and PBMCs. **B** Gating of CD45^+^ CD8^+^ T cells and gating of CD4^+^ FOXP3^+^ Treg cells in the PBMCs of the coculture system. **C** The experiment was repeated nine times, and the differences between the groups were analyzed by two-way analysis of variance. The values are expressed as the means ± SDs. *P ≤ 0.05. NC: PBMCs without coculture, CO1: PBMCs cocultured with untreated macrophages, CO2: PBMCs cocultured with CSF-1-induced macrophages, CO3: PBMCs cocultured with CSF-1-induced macrophages pretreated with the anti-CSF-1R antidoby, and CO4: PBMCs were cocultured with CSF-1-induced macrophages pretreated with the anti-DC-SIGN antibody

On the basis of the successful induction of macrophage differentiation from THP-1 cells, we established a coculture system with macrophages and peripheral blood lymphocytes (Fig. 5A). Lymphocytes express higher levels of the CD45 antigen, which is exclusively expressed on all nucleated cells of the hematopoietic system, and the different proportions of the monocyte/macrophage populations in the PBMC coculture system can be further confirmed in morphological cytograms showing the FSC (forward scatter) and SSC (side scatter) parameters obtained by flow cytometry (Fig. 5B). The number of CD8-positive T cells among the PBMCs in the CO2 group (treated with only CSF-1) was significantly lower than that in the NC group (not cocultured) and CO3 group (treated with both CSF-1 and the anti-CSF-1R antibody), while the number of Treg cells among the PBMCs in the CO2 group (treated with only CSF-1) was significantly higher than that in the NC group (not cocultured) and CO3 group (treated with both CSF-1 and the anti-CSF-1R antibody) (Fig. 5B). Interestingly, there were no differences in the numbers of CD8-positive T cells and Treg cells among the PBMCs in the CO4 group (treated with both CSF-1 and the anti-DC-SIGN antibody), CO2 group (treated with only CSF-1), NC group (not cocultured) and CO3 group (treated with both CSF-1 and the anti-CSF-1R antibody) (Fig. 5B). Similarly, there were no differences in the numbers of CD8-positive T cells and Treg cells among the PBMCs in the CO1 group (cocultured only), CO2 group (treated with only CSF-1), NC group (not cocultured) and CO3 group (treated with both CSF-1 and the anti-CSF-1R antibody) (Fig. 5B). Through data analysis, we clearly found that the DC-SIGN^+^ macrophages induced by CSF-1 led to a depressed phenotype of peripheral blood lymphocytes, while the CSF-1 antagonist anti-CSF-1R abrogated this biological effect (Fig. 5C).

## Discussion

CSF-1 is a cytokine that induces monocytes to differentiate into CD169^+^ macrophages [14]. Our study was consistent with this observation, and we found that the CSF-1 antagonist anti-CSF-1R abolished this effect. Additionally, we found that CSF-1 induced monocytes to differentiate into DC-SIGN^+^ macrophages; similarly, anti-CSF-1R abolished this effect. Moreover, we found that DC-SIGN^+^ macrophages led to a suppressive phenotype of peripheral blood lymphocytes, including a high percentage of Treg cells and a low percentage of CD8^+^ T cells, which was agreement with previous study [20]; and this biological effect can be abrogated by anti-CSF-1R.

DC-SIGN is expressed in human DCs and macrophages [28, 37]. Importantly, DC-SIGN is involved in multiple aspects of the immunological response [38]; Yvette van Kooyk’s laboratory proposed that DC-SIGN can actively contribute to the maintenance of an immunosuppressive tissue environment [39]; as previously described, CD169^+^ macrophages, which are known as CD169^+^ suppressive macrophages [18], play a role in various diseases; but the role of DC-SIGN^+^ CD169^+^ macrophages in the pathogenesis of endometriosis has not been studied. We found that the proportion of CD169^+^ DC-SIGN^+^ macrophages was higher in the PF of endometriosis patients; this phenomenon was not in agreement with observations in tissues. Although DC-SIGN^+^ macrophages were more abundant in the ectopic endometrium group than in the eutopic endometrium and the normal endometrium groups, the number of CD169^+^ macrophages was not different among the three groups. These differences may be due to the diversity of phenotypic differentiation of macrophages in different tissues and regions.

We also found that macrophages coexpressing CSF-1R and CD169 were more abundant in the PF of endometriosis patients than in that of controls. A previous study proved that CSF-1R inhibition altered macrophage polarization and blocked glioma progression [40]. CSF-1R and its ligand, CSF-1, are expressed diffusely and have been frequently reported in patients with EMS [15, 16]. However, in the tissue of ectopic lesions, CSF-1R expression did not differ from that in the normal endometrium and the eutopic endometrium of endometriosis patients. This result is not consistent with a previous study in which CSF-1R was found in nearly 70% of endometriosis patients and was found at a statistically significantly lower percentage in normal endometrium [41], hence this difference needs to be defined in more studies.

Although the protein expression level of CSF-1R in the ectopic lesions of endometriosis patients was not different from that in the eutopic endometrium of endometriosis patients and the normal endometrium, the gene expression level of CSF-1 was significantly higher in these lesions. The level of the CSF-1 cytokine in the PF of endometriosis patients was higher than that in the PF of controls. Our in vivo experiment showed that the peritoneum of all mice contained high levels of the cytokine CSF-1, while the level of the cytokine CSF-1 was significantly higher in the ectopic endometrial tissue of mice with endometriosis than in the eutopic endometrial tissue of mice with endometriosis and the normal endometrium of control mice. The principal source of CSF-1 may be ectopic lesions in mice with endometriosis. This result was in agreement with a previous study that proved that compared with eutopic tissue, ectopic endometrial tissue exhibited a 3.5-fold increase in CSF-1 expression [15]. Therefore, CSF-1 may be an important cytokine that induces monocytes to differentiate into CD169^+^ DC-SIGN^+^ suppressive macrophages.

## Conclusions

Overall, CSF-1 and its receptor, CSF-1R, are important for the induction of CD169^+^ DC-SIGN^+^ macrophages. We also demonstrate here that CSF-1-induced DC-SIGN^+^ macrophages inhibit the increase in CD8^+^ T cells and the decrease in Treg cells. We are studying the detailed mechanism by which DC-SIGN^+^ macrophages affect the proliferation and differentiation of T cells in EMS. This study provides a framework for developing CSF-1-based in vitro protocols to generate therapeutic macrophages for clinical use to regulate the immune environment of endometriosis.

## Data Availability

The datasets used and analyzed during the present study are available from the corresponding author on reasonable request.
